# Proteoglycans Are Attractive Biomarkers and Therapeutic Targets in Hepatocellular Carcinoma

**DOI:** 10.3390/ijms19103070

**Published:** 2018-10-08

**Authors:** Yasuo Tanaka, Ryosuke Tateishi, Kazuhiko Koike

**Affiliations:** Graduate School of Medicine, Department of Gastroenterology, The University of Tokyo, 7-3-1, Hongo, Bunkyo-ku, Tokyo 113-8655, Japan; tateishi-tky@umin.ac.jp (R.T.); kkoike-tky@umin.ac.jp (K.K.)

**Keywords:** proteoglycan, hepatocellular carcinoma, serglycin, syndecan-1, glypican 3, agrin, collagen XVIII, endostatin, versican, decorin

## Abstract

Proteoglycans, which consist of a protein core and glycosaminoglycan chains, are major components of the extracellular matrix and play physiological roles in maintaining tissue homeostasis. In the carcinogenic tissue microenvironment, proteoglycan expression changes dramatically. Altered proteoglycan expression on tumor and stromal cells affects cancer cell signaling pathways, which alters growth, migration, and angiogenesis and could facilitate tumorigenesis. This dysregulation of proteoglycans has been implicated in the pathogenesis of diseases such as hepatocellular carcinoma (HCC) and the underlying mechanism has been studied extensively. This review summarizes the current knowledge of the roles of proteoglycans in the genesis and progression of HCC. It focuses on well-investigated proteoglycans such as serglycin, syndecan-1, glypican 3, agrin, collagen XVIII/endostatin, versican, and decorin, with particular emphasis on the potential of these factors as biomarkers and therapeutic targets in HCC regarding the future perspective of precision medicine toward the “cure of HCC”.

## 1. Introduction

Hepatocellular carcinoma (HCC) is among the most common cancers worldwide and the leading cause of cancer-related deaths, especially in less economically developed regions [1]. HCC is highly resistant and refractory to therapeutic interventions, such as surgical resection or radiofrequency ablation (RFA) therapy. In our institution, the 5- and 10-year survival rates of HCC patients treated with RFA were 60.2% and 27.3%, respectively [2]. One of the reasons for this is that the carcinogenic tissue microenvironment in the liver can give rise to recurrent de novo HCC tumors; therefore, it is almost impossible to “cure HCC”.

The liver microenvironment consists of several components including the extracellular matrix (ECM), immune cells, Kupffer cells, endothelial cells, fibroblasts, cytokines, and various growth factors [3]. Proteoglycans are one of the major components of the ECM; they contain at least one glycosaminoglycan chain (heparan sulfate, chondroitin sulfate, keratan sulfate, and heparin) that is covalently attached to the core protein. Proteoglycans are classified based on three criteria: Cellular and subcellular location (intracellular, cell surface, pericellular, and extracellular), overall gene/protein homology, and the presence of specific protein modules within the protein cores (Table 1) [4].

In healthy tissues, proteoglycans are essential for proper structural development, organization, and hydration and also exert functional effects by interacting with other matrix structures, cells, and cellular mediators. In a carcinogenic tissue microenvironment, the expression of proteoglycans is altered markedly. Modified proteoglycan expression on tumor and stromal cell membranes affects cancer cell signaling, resulting in changes in growth, migration, and angiogenesis that facilitate tumorigenesis by modulating proteoglycan function [5].

Although more than three dozen proteoglycans have been discovered, physiological roles in the liver have only been investigated for a small number. In addition, the number of proteoglycans of which the function and precise mechanisms of action are understood is very limited.

Herein, we provide an overview of the current understanding of proteoglycans in HCC development and progression, with a particular focus on well-investigated proteoglycans, including serglycin, syndecan-1, glypican 3, agrin, collagen XVIII/endostatin, versican, and decorin. Among them, glypican 3 is the most promising molecule for both diagnosis and therapeutic applications in HCC, and follow syndecan-1 and collagen XVIII/endostatin. We also discuss the potential of proteoglycans as biomarkers and therapeutic targets for HCC to obtain an opportunity for much more precise personalized patient care in this “precision medicine” era.

## 2. Intracellular Proteoglycan: Serglycin

Serglycin is a proteoglycan with a small protein core (158 amino acids (aa) in humans). It is unique since it is the only intracellular proteoglycan discovered to date and is covalently substituted with heparin as a glycosaminoglycan in its consecutive Ser-Gly repeats [4]. Serglycin is mainly expressed in hematopoietic lineage cells and contributes to the proper storage and secretion of inflammatory mediators such as proteases, histamine, cytokines, and chemokines [6]. Recent studies have demonstrated that serglycin is overexpressed in a variety of cancers, including colon, breast [7], and lung [8] cancers, and that serglycin overexpression was correlated with a more aggressive malignant phenotype. In the normal liver, serglycin is weakly expressed mainly in the cytoplasm. In contrast, serglycin levels were significantly upregulated in HCC tissues, which was positively associated with vascular invasion, advanced Barcelona clinic liver cancer (BCLC) staging, early recurrence, and unfavorable prognosis in HCC patients. Furthermore, serglycin expression levels were an independent predictor of overall survival and time to recurrence in HCC patients, suggesting that it could predict a malignant phenotype similar to other types of cancer [9].

Although serglycin is an intracellular proteoglycan, it can also be secreted into serum or incorporated into the ECM [10]. Serglycin levels have been shown to be increased in the sera of HCC patients with bone metastasis. A diagnostic model established with serglycin and six other peptides (α-fetoprotein, prothrombin, isoform 2 of inter-alpha-trypsin inhibitor heavy chain H4, isoform 1 of autophagy-related protein 16-2, and transthyretin and fibrinogen beta chains) achieved a high recognition rate and predictive power for HCC patients with bone metastasis. Therefore, these serum peptides might serve as a diagnostic tool for HCC bone metastasis [11], although this requires further validation.

Mechanistically, serglycin can activate several cancer-associated signaling pathways in other types of cancer, including mitogen-activated protein kinase (MAPK)/β-catenin signaling [12], transforming growth factor-β2 (TGF-β2) signaling via interaction with CD44 [13], and the NF-κB pathway [8]. However, the function of serglycin in the liver is not well understood and needs to be investigated further.

## 3. Cell Surface Proteoglycan (Transmembrane): Syndecan-1/CD138

Syndecans are a major family of cell surface heparan sulfate proteoglycans. The syndecan family consists of four distinct genes (*syndecan-1*, *-2*, *-3*, and *-4*) encoding single transmembrane protein cores that include an ectodomain, a transmembrane region, and an intracellular domain [4]. Syndecan-1 was the first discovered syndecan [14] and has been well investigated. The protein core of syndecan-1 is 310 aa long in humans and is widely expressed in epithelial cells.

In normal liver tissues, syndecan-1 expression was observed in sinusoids. As cirrhosis progresses, syndecan-1 expression is increased and its localization extended to the entire hepatocyte membrane surface. Although one study reported that syndecan-1 is strongly expressed in the cell membrane of HCC tissues [15], another report showed that approximately 68% of HCC samples exhibited negative staining for syndecan-1 and that its expression was significantly reduced in poorly differentiated HCC and extra-hepatic metastasis [16]. Similarly, another study reported that positive syndecan-1 protein expression in HCC was associated with good differentiation and no extrahepatic metastasis [17]. These results suggest that loss of syndecan-1 expression is a characteristic of poorly differentiated HCC with high metastatic potential.

In addition to the transmembrane form, syndecans can also be found in a soluble form that has shed from the cell surface. The shedding of syndecan-1 occurs at a specific juxtamembrane cleavage site; cleavage is performed by various matrix metalloproteinases (MMPs), including MMP-7 [18]. Syndecan-1 levels in serum increased with the progression of fibrosis in chronic hepatitis C patients [19]. Furthermore, the serum levels of syndecan-1 were increased significantly in HCC patients compared with cirrhotic patients, which was associated with increasing BCLC staging [20]. Consistent with this, among patients with alcoholic cirrhosis, high serum syndecan-1 levels were significantly associated with a greater risk of tumor recurrence in patients with early HCC treated with RFA, as well as with less favorable overall survival [21]. This suggests that the increase in serum syndecan-1 levels may be linked with both fibrosis and the progression of HCC. Overall, these results suggest that syndecan-1 is overexpressed in HCC; however, it is also shed by a protease and found at high concentrations in serum, which could reconcile the low expression of syndecan-1 found in HCC cell membranes and the high concentrations found in the serum of HCC patients.

The shedding of syndecan-1 is useful as a biomarker but also has biological significance as the shed ectodomain can bind to other ECM components or compete with membrane-bound syndecan-1 for ligand binding to modulate its biological functions [22]. A recent study showed the importance of shedding syndecan-1 in the epithelial-mesenchymal transition (EMT) in HCC [23]. Sphingosine-1-phosphate (S1P) induced the EMT in HCC via an unknown mechanism [24]. The authors of this study found that high serum S1P levels in HCC patients were positively correlated with serum syndecan-1 levels, whereas the opposite correlation was observed with syndecan-1 and S1P. S1P activates the PI3K/Akt signaling pathway via the sphingosine 1-phosphate receptor (S1P1), which triggers heparanase expression and increases the expression and activity of MMP-7 as well as syndecan-1 shedding. In HCC, the loss of syndecan-1 resulted in increased TGF-β1 production and induced the EMT in HCC via an MMP-7/syndecan-1/TGF-β autocrine loop. Heparanase is an endoglucuronidase that cleaves heparan sulfate chains of proteoglycans, and high heparanase expression and activity have been correlated with an aggressive tumor phenotype in HCC [25]. Preclinical and clinical studies have demonstrated that therapies targeting the heparanase /syndecan-1 axis are promising for blocking aggressive cancer phenotypes [26], which could lead to HCC treatments in the future.

Syndecan-1 forms a complex with insulin-like growth factor 1 receptor (IGF-1R) and αvβ3 integrin and plays an important role in angiogenesis and tumorigenesis. This complex is highly expressed on tumor cells and is activated in endothelial cells during angiogenesis. Clinically, a selective peptide inhibitor of the syndecan-1-IGF-1R-αvβ3 integrin complex, synstatin, was developed to competitively displace IGF-1R and integrin from syndecan and inactivate the complex [27,28]. A preclinical study of synstatin using a rat HCC model showed that inhibiting HCC in vivo by downregulating the integrin αvβ3 receptor reduced activation of the angiogenic growth factors, vascular endothelial growth factor (VEGF), and basic fibroblast growth factor (bFGF/FGF-2) [29], suggesting that this could be a promising targeted therapy in HCC.

## 4. Cell Surface Proteoglycan (Glycosylphosphatidylinositol-Anchored): Glypican-3

Glypicans (GPC) are heparan sulfate proteoglycans that bind to the plasma membrane through their C-terminal glycosylphosphatidylinositol (GPI) anchor. The GPC family consists of six distinct genes (*GPC1–6*) that are all highly expressed during embryonic development.

GPC3 gained attention when a study used cDNA microarray analysis to reveal that it was highly expressed in HCC but not expressed in the normal liver [30], which was confirmed by subsequent reports [31,32]. Moreover, since GPC3 was detected in HCC cells but not in benign liver tumors such as dysplastic nodules, it has potential as a biomarker for the diagnosis of early stage HCC [33,34,35]. The combination of GPC3 with heat shock protein-70 and glutamine synthetase could be used for the diagnosis of HCC to distinguish small, well-differentiated HCC from dysplastic nodules, and the use of two of these markers increased the specificity of early HCC diagnosis [36,37].

Many studies have reported that GPC3 expression is correlated with a poor prognosis in HCC [38,39], and that GPC3 may be a prognostic marker for curative resection [40,41,42] and HCC recurrence following liver transplantation [43]. Two meta-analyses recently indicated that GPC3 overexpression was significantly associated with poor prognosis in patients with HCC [44,45].

GPC3 can also be detected in serum. The NH_2_-terminal portion of GPC3 is cleaved between Arg-358 and Ser-359 to generate soluble GPC3, which is specifically detected in the sera of patients with HCC [46]. GPC3 can be cleaved by the α/β-hydrolase enzyme Notum [47], releasing the N-terminal domain and full-length GPC3 from the cell surface; this could be the mechanism through which GPC3 is secreted into serum.

As a tumor marker, GPC3 was detected in the serum of 40% of HCC patients, but not in cirrhotic patients [48]. Furthermore, among HCC patients who were seronegative for both alpha-fetoprotein (AFP) and des-gamma-carboxy prothrombin (DCP), one-third were positive for GPC3. However, no correlation between GPC3 and AFP was observed [34], suggesting that GPC3 could complement the diagnosis of HCC by functioning as a tumor marker. In a meta-analysis, the pooled sensitivity and specificity of serum GPC3 for the diagnosis of HCC was comparable to AFP, and the sensitivity of the HCC diagnosis was increased if GPC3 was combined with AFP. However, the diagnostic accuracy of serum GPC3 for early HCC was not satisfactory [49].

Mechanistically, GPC3 inhibits hedgehog protein signaling during development by competing with the hedgehog receptor, patched, for hedgehog binding [50]. GPC3-deficient mice exhibited developmental overgrowth and some of the abnormalities typical of Simpson-Golabi-Behmel syndrome (SGBS), which is a rare X-linked disorder in males carrying *GPC3* mutations. SGBS is characterized by pre/postnatal overgrowth, developmental delay, macrocephaly, characteristic facial features, diaphragmatic hernia, congenital heart defects, and kidney and skeletal anomalies [51]. The overgrowth observed in SGBS patients is considered, at least in part, a consequence of hyperactivation of the hedgehog signaling pathway, via GPC3 acting as a negative regulator of Hedgehog signaling through interaction with Sonic hedgehog (SHH) [50]. Although a recent study showed that GPC3 may promote HCC proliferation through the hedgehog pathway in vitro [52], the role of GPC3 in the inhibition of hedgehog signaling during HCC development remains controversial.

GPC3 also stimulates the canonical Wnt signaling pathway by directly interacting with Wnts and Frizzled to promote the growth of HCC [53,54]. Dysregulation of the Wnt/β-catenin pathway is an early event in hepatocarcinogenesis and is associated with an aggressive HCC phenotype [55], suggesting that GPC3 is a potential therapeutic target to prevent the genesis and progression of HCC.

GPC3 also interacts with insulin-like growth factor (IGF)-II and IGF-1R and stimulates the phosphorylation of IGF-1R and the downstream signaling molecule extracellular signal-regulated kinase (ERK) [56], which might contribute to the EMT in HCC [57].

The potential of GPC3 is not limited to its use as a serum biomarker. As GPC3 is upregulated exclusively in HCC, it has been used as an immune-specific target for cancer immunotherapy. GC33, a recombinant fully humanized monoclonal antibody that binds to human GPC3, has been shown to exert antibody-dependent cell-mediated cytotoxicity against GPC3-positive human HCC cells in vitro and in vivo [58,59] in a preclinical setting. A phase I clinical trial was performed in the USA and Japan, which revealed good tolerance and moderate antitumor effects [60,61]. An international phase II placebo-controlled trial is ongoing.

GPC3 peptides are also in development as a cancer vaccine. This peptide vaccine is restricted to human leukocyte antigen (HLA)-A24 and HLA-A2, which are present in ~60% and 40% of Japanese individuals, respectively; the latter is also a major haplotype in Caucasians. Mice immunized with GPC3 peptides develop antigen-specific cytotoxic T lymphocytes (CTLs) that exhibit antitumor activity [62,63,64]. A phase I clinical study of the GPC3 peptide vaccine in patients with advanced HCC was performed, which revealed it was well-tolerated and that the vaccine induced a GPC3-specific CTL response in approximately 90% of patients; subjects with a high level of CTLs had better overall survival [65]. Subsequently, a phase II study assessing the GPC3 peptide vaccine as an adjuvant treatment for HCC following surgical resection or RFA was performed [66]. Although the 1- and 2-year recurrence rates were 24.4% and 53.7%, respectively, and the primary endpoint was not reached, the GPC3 peptide vaccine improved the 1-year recurrence rate in patients with GPC3-positive tumors. Therefore, GPC3 expression in the primary tumor could be used as a biomarker to determine the efficacy of GPC3-derived peptide vaccines.

A recently developed promising therapy for HCC is a chimeric antigen receptor (CAR)-T cell therapy that engineered the expression of CARs on the surface of T cells to redirect T-cell specificity to target cancer [67]. Using an HCC xenograft model in mice, T cells expressing the third-generation GPC3-targeted CAR could not only kill HCC cells expressing high levels of GPC3, but also efficiently suppressed the growth of HCC expressing low levels of GPC3 in vivo [68]. An approach that could reduce the risk of on-target, off-tumor toxicity while maintaining relatively potent antitumor activity could improve this attractive therapy [69,70].

## 5. Pericellular Proteoglycan: Agrin

The heparan sulfate proteoglycan agrin is best known as a crucial organizer of postsynaptic differentiation at the neuromuscular junction [71], and agrin mutant mice die around birth due to disrupted neuromuscular function [72]. In spite of its functions in the establishment and maintenance of neuromuscular junctions, the role of agrin outside the neuromuscular junction is poorly understood.

In the normal liver, agrin was observed around bile ducts and blood vessels within portal areas, but not within hepatic lobules [73,74]. Agrin staining was also negative in benign tumors such as hepatocellular adenoma, dysplastic nodules, and large regenerative nodules [75]. In contrast, agrin expression is elevated dramatically in chronic liver disease and HCC, where it is deposited in the vascular and peribiliary basement membranes [73].

The role of agrin in HCC progression is unknown. However, a recent report found that multiple HCC cell lines highly express and secrete agrin [76]. A study used stable isotope labeling using amino acids in cell culture (SILAC) quantitative proteomics to demonstrate that agrin was overexpressed in HCC cell line samples of cell surface proteins enriched for plasma membrane fractions. The overexpression of secreted and cell surface agrin increases binding to its neuronal receptor machinery (Low-Density Lipoprotein (LDL)-receptor related protein 4 Lrp4/muscle-specific tyrosine kinase MuSK) and promotes the formation of the agrin–Lrp4/MuSK signaling complex. Then, this complex functions as an ECM sensor and activates focal adhesion kinase, which is essential for hepatic tumorigenesis.

Agrin is secreted from human hepatic stellate cells activated by platelet-derived growth factor (PDGF) and induces the EMT in HCC cells [77]. Interestingly, the multi-kinase inhibitor sorafenib, which targets the PDGF receptor (PDGFR), not only inhibits the hepatocarcinogenesis mediated through agrin secretion, but also alleviates liver inflammation and fibrosis, suggesting that it might be a potential candidate for the treatment of cirrhosis.

## 6. Pericellular Proteoglycan: Collagen XVIII/Endostatin

Collagen XVIII is a large basement membrane heparan sulfate proteoglycan that is ubiquitously expressed. There are three variants of collagen XVIII: Short (1336 aa), middle (1516 aa), and long (1751 aa) [78]. The short isoform is found in the human heart, kidney, placenta, ovary, skeletal muscle, and small intestine [79]. By contrast, the long form is found almost exclusively in the liver; it is expressed at remarkably high levels by normal human hepatocytes and regulated by liver-enriched transcription factors including HNF3β/FOXA2 [80]. In the normal liver, type XVIII collagen, which is mainly produced by hepatocytes, is heavily deposited in perisinusoidal spaces and the basement membrane. In cirrhosis, it is derived from activated stellate cells and forms a thick deposit along capillarized sinusoids [81,82]. However, one report showed that collagen XVIII is downregulated in HCC and is associated with HCC recurrence within 2 years of resection, which may need further validation [83].

Interestingly, the C-terminus of collagen XVIII is cleaved by various proteases, producing a 20-kDa fragment called endostatin [84,85]. Endostatin acts as a powerful anti-angiogenic agent by interfering with the pro-angiogenic actions of growth factors such as VEGF [86] and bFGF/FGF-2 [87].

As a biomarker, increased endostatin/collagen XVIII expression was correlated with elevated VEGF levels and a poor prognosis in HCC [88]. In contrast, another report showed that patients with high serum endostatin levels in their preoperative sample had significant low-vascularity cancer and a tendency toward long survival [89]. In addition, serum endostatin levels were reported to fail to have a significant prognostic influence on overall or disease-free survival [90], which suggests that endostatin levels in serum may reflect the tumor burden rather than antiangiogenic activity in the tumor [91].

As an anti-angiogenic treatment, gene therapy using a vector expressing endostatin exerted anti-tumor effects in the preclinical setting [92,93,94,95]. To apply endostatin itself as an anti-angiogenic therapy, recombinant human endostatin (Endostar), expressed and purified in *Escherichia coli*, was developed and approved by the State Food and Drug Administration of China in 2005 [96]. It was shown to promote the efficiency of chemotherapy during the treatment of advanced non-small cell lung cancer phase II/III clinical trials [97]. Preclinical studies of Endostar also showed anti-angiogenic effects in HCC in vitro [98] and in vivo [99], suggesting that combination therapy with Endostar could be considered for the treatment of HCC.

## 7. Extracellular Proteoglycan (Hyalectan): Versican

Versican is a large chondroitin sulfate proteoglycan that interacts with hyaluronan via specific domains in its core protein. The N-terminal globular domain (G1 domain) of versican contains an immunoglobulin-like motif and two proteoglycan tandem repeats that bind to hyaluronan. The C-terminal globular domain (G3 domain) contains two Epidermal Growth Factor (EGF)-like repeats, a complement regulatory protein-like repeat, and a C-type lectin domain [100]. The sizes of the four versican isoforms V0, V1, V2, and V3 are 370, 263, 180, and 74 kDa, respectively. These isoforms differ by the presence or absence of two glycosaminoglycan (GAG) attachment regions, GAG-α and GAG-β. The V0 isoform contains both GAG-α and GAG-β, whereas V1 contains GAG-β, V2 contains GAG-α, and V3 contains no GAG attachment domains. Although V0, V1, and V3 are expressed ubiquitously, the expression of V2 is mostly restricted to the central nervous system [101].

In cirrhotic livers, versican expression increases, which is partially perisinusoidal and stromal but substantially cytoplasmic in hepatocytes [102,103]. In HCC, versican expression was significantly upregulated compared with adjacent nontumor tissues and mainly localized in the cytoplasm [104,105]. Quantitative tissue proteomics comparing HCC and corresponding tissue samples revealed that versican core protein was significantly abundant in well-differentiated and early-stage HCC, suggesting that versican is a potential biomarker for early-stage HCC [106].

Versican regulates a variety of cell activities, including adhesion, proliferation, apoptosis, migration, and invasion, via the chondroitin and dermatan sulfate side chains and the G1 and G3 domains [107,108,109,110] by interacting with hyaluronan and forming large aggregates through the G1 domain or activating Epidermal Growth Factor Receptor (EGFR) signaling through EGF-like motifs in the G3 domain. Each isoform of versican has distinct functions. V1 has cancer-promoting functions such as enhancing cell proliferation, inducing apoptosis resistance, inhibiting cell adhesion, and promoting cell motility [111,112,113]. A recent study demonstrated that versican acts on macrophages through the toll-like receptors TLR2 and TLR6, leading to the production of inflammatory cytokines and the promotion of tumor cell metastasis [114].

*Versican* transcription is regulated by p53 [115], AP-1, and T-cell factor (TCF) [116] in a cell type-specific manner. In HCC, versican is partially regulated by the Wnt/β-catenin pathway together with Sharpin. Sharpin is a component of the linear ubiquitin chain assembly complex, which is upregulated in various types of cancer including HCC [117]. To understand the molecular mechanisms through which Sharpin modulates oncogenesis, we performed cDNA microarray analysis and found that versican is upregulated in Sharpin-expressing cells [105]. Sharpin interacts with endogenous β-catenin and synergistically increases *versican* promoter activation by activating the Wnt/β-catenin pathway, possibly as a coactivator of TCF/LEF. This suggests that Sharpin cooperates with the activated Wnt/β-catenin pathway to recruit β-catenin to the *versican* promoter region and stabilize the interaction, thereby inducing versican transactivation and HCC invasion [118].

In HCC, versican V1 was also transactivated by the transcription factor FoxQ1, which induced the EMT by inducing secretion of chemokine (C–C motif) ligand 2 (CCL2) from cancer cells and macrophage infiltration in HCC. This was associated with poorer prognosis [104]. Recent studies showed that versican expression is also regulated by various microRNAs (miRNAs) [119,120]. Moreover, the *versican* 3’-untranslated region (3’-UTR) modulates endogenous miRNA functions by acting as a competitive endogenous RNA [121]. Interestingly, transgenic mice expressing the *versican* 3’-UTR expressed high levels of the versican isoforms V0 and V1 and develop HCC, suggesting that the *versican* 3’-UTR functions as a competitive endogenous RNA to induce the development of HCC by regulating miRNA activity to ensure high versican V1 expression and hepatocarcinogenesis [122].

## 8. Extracellular Proteoglycan (Small Leucine-Rich Proteoglycans): Decorin

Decorin is a small cellular or pericellular matrix proteoglycan that belongs to the small leucine-rich proteoglycan family. It is most abundant in the skin, connective tissues, muscles, and kidney [4]. In the healthy liver, decorin levels are generally low. However, decorin accumulates in chronic liver injury and is deposited along the sinusoidal walls [123]. Interestingly, decorin colocalizes with large amounts of TGF-β1, a key stimulator of fibrillogenesis and fibrogenesis, in fibrotic areas in chronic hepatitis and cirrhosis [124]. Decorin blocks signaling through TGF-β and modulates degradation of the ECM by inducing expression of the MMP collagenase-1 [125,126]. In HCC, *decorin* gene knockout enhanced experimental hepatic fibrosis and impaired the healing of hepatic fibrosis in mice [127]. Therapeutically, in a human hepatic stellate cell line, decorin inhibited TGF-β signaling, downregulated α-smooth muscle actin expression, and decreased cell proliferation [128].

Decorin acts as a tumor suppressor in a variety of cancers, mainly by blocking the action of receptor tyrosine kinases such as the receptors for hepatocytes and epidermal and insulin-like growth factors. In a model of chemical carcinogen-induced HCC, *decorin* knockout mice exhibited enhanced tumor prevalence and higher tumor count compared with wild-type mice [129]. Mechanistically, PDGFRα, EGFR, IGF-IR, and macrophage-stimulating 1 receptor (MST1R) were activated in *decorin*-deficient mice, suggesting that decorin acts as a secreted tumor suppressor during hepatocarcinogenesis by hindering the action of another receptor tyrosine kinase. Therefore, decorin could be a novel antitumor agent in HCC.

## 9. Future Perspectives

**Figure 1:**
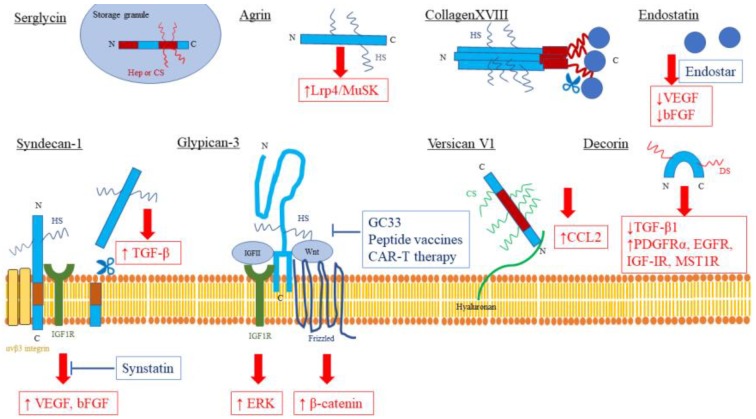
Proteoglycans as therapeutic targets in liver diseases. Therapeutic targets are highlighted in red boxes, and developing therapies are highlighted in blue boxes. N: amino-terminal, C: Carboxy-terminal, HS: Heparan Sulfate, CS: Chondroitin Sulfate, DS: Dermatan Sulfate, ↑: activated, ↓: inactivated, T-bar: inhibitor.

As discussed in this review, proteoglycans are ECM components that play a crucial role in the progression of HCC and have received significant attention as potential biomarkers and therapeutic targets (summarized in Table 2 and Table 3 and Figure 1.). Although proteoglycans have been investigated extensively and a large amount of knowledge regarding their roles in HCC has been collected, prognostic (recurrence, metastasis, and overall survival) or predictive (response to specific therapy) markers are still not available and only a few clinical trials targeting GPC3 are ongoing.

President Obama’s Precision Medicine Initiative was launched on 30 January 2015 [130]; however, it is still far away from achieving precision medicine targeting proteoglycans. One hope is that, as mentioned in Section 4 above, GPC3 could be an attractive target antigen for CAR-T cell therapy. A recent study showed that chondroitin sulfate proteoglycan 4 (CSPG4) is also an attractive target antigen for glioblastoma [131]. Intratumoral heterogeneity of proteoglycan expression may promote tumor immune escape due to antigen loss in this kind of therapy. Furthermore, CAR-T cell therapy-associated toxicities, including cytokine-release syndrome, CAR-T-cell-related encephalopathy syndrome, and fulminant hemophagocytic lymphohistiocytosis need to be well monitored and managed [132]. This novel immunotherapy targeting proteoglycans overexpressed in malignant tumors has powerful potential to help attain complete responses to different types of cancer, including HCC.

Novel therapeutic approaches that take into account individual differences in “proteoglycans” expression may pave the way for the realization of “precision medicine” to cure this currently untreatable cancer.

## Figures and Tables

**Table 1:** A comprehensive classification of proteoglycans. (modified from Reference [4], Reproduced with permission from Iozzo, R.V. et al., Matrix Biology; published by Elsevier, 2015).

Location	Classification	Eponym	Predominant GAG
Intracellular	Secretory granules	Serglycin	Hep
Cell surface	Transmembrane	Syndecan, 1–4	HS
		NG2	CS
		Betaglycan	CS/HS
		Phosphacan	CS
	GPI-anchored	Glypican, 1–6	HS
Pericellular	Basement membrane zone	Perlecan	HS
		Agrin	HS
		Collagen XVIII	HS
		Collagen XV	CS/HS
Extracellular	Hyalectan Lectican	Aggrecan	CS/KS
		Versican	CS
		Neurocan	CS
		Brevican	CS
	SLRPs:canonical class I	Biglycan	CS
		Decorin	DS
		Asporin	
		ECM2	
		ECMX	
	SLRPs:canonical class II	Fibromodulin	KS
		Lumican	KS
		PRELP	
		Keratocan	KS
		Osteoadherin	KS
	SLRPs:canonical class III	Epiphycan	DS/CS
		Optican	
		Osteoglycin	
	SLRPs:non-canonical class VI	Chondroadherin	
		Nyctalopin	
		Tsukushi	
	SLRPs:non-canonical class V	Podocan	
		Podocan-Like 1	
	SPOCK	Testican, 1–3	HS

GAG: Glycosaminoglycan, Hep: Heparin, HS: Heparan Sulfate, CS: Chondroitin Sulfate, KS: Keratan Sulfate, DS: Dermatan Sulfate, GPI: Glycosylphosphatidylinositol, SLRPs: Small leucine-rich proteoglycans. The proteoglycans that are discussed in this review are in red.

**Table 2:** Proteoglycans as biomarkers in liver diseases.

Proteoglycan	Sample	Biomarker
**Serglycin**	**Tissue**	↑ HCC with vascular invasion, advanced BCLC staging, and unfavorable prognosis [9]
	**Serum**	↑ HCC with bone metastasis [11]
**Syndecan-1**	**Tissue**	↓ HCC with poor differentiation and high metastatic potential [16,17]
	**Serum**	↑ CLD with fibrosis [19] ↑ HCC with advanced BCLC staging [20] ↑ HCC recurrence [21]
Glypican-3 *	**Tissue**	↑ HCC with poor prognosis [38,39] ↑ HCC recurrence after operation [40,41,42] ↑ HCC recurrence after liver transplantation [43]
	**Serum**	↑ HCC (Independent to AFP) [34,48]
**Agrin**	**Tissue**	↑ CLD and HCC [73]
	**Serum**	N/A
**CollagenXVIII/Endostatin**	**Tissue**	N/A
	**Serum**	N/A
**Versican**	**Tissue**	↑ CLD with fibrosis [102,103,105] ↑ HCC with poor prognosis [104,105] ↑ early-stage HCC [106]
	**Serum**	↑ CLD with fibrosis [103]
**Decorin**	**Tissue**	↑ CLD with fibrosis [123]
	**Serum**	N/A

BCLC: Barcelona clinic liver cancer, CLD: Chronic liver disease, N/A: Not available, *: Applied for diagnosis, ↑: increased, ↓: decreased.

**Table 3:** Proteoglycans as therapeutic targets in liver diseases.

Proteoglycan	Therapeutic Target and Agent	
**Serglycin**	**Therapeutic target**	N/A
	**Agent**	N/A
Syndecan-1 *	**Therapeutic target**	↑ MMP-7/syndecan-1/TGF-β [23] ↑ Syndecan-1-IGF1R-αvβ3 integrin complex / VEGF, bFGF [27,28,29]
	**Agent**	Synstatin (Syndecan-1-IGF1R-αvβ3 integrin complex inhibitor) (Preclinical) [27,28,29]
Glypican-3 **	**Therapeutic target**	↑ Wnt/Frizzled/β-catenin [53,54] ↑ IGF-II/IGF-1R/ERK [56,57] ↓ SHH/Patched-1 [52]
	**Agent**	GC33(recombinant humanized monoclonal antibody) (Phase II) [60,61] Peptide vaccines (Phase II) [65,66] CAR-T therapy (preclinical) [68,69,70]
**Agrin**	**Therapeutic target**	↑ Lrp4/MuSK [76]
	**Agent**	N/A
CollagenXVIII/Endostatin *	**Therapeutic target**	↓ VEGF [86] ↓ bFGF/FGF-2 [87]
	**Agent**	Gene therapy (preclinical) [92,93,94,95] Endostar (Recombinant human endostatin) (preclinical) [98,99]
**Versican**	**Therapeutic target**	↑ Versican/CCL2 [104]
	**Agent**	N/A
**Decorin**	**Therapeutic target**	↓ TGF-β1 [125,126] ↑ PDGFRα, EGFR, IGF-IR, MST1R [129]
	**Agent**	N/A

N/A: not available. CAR-T therapy: A chimeric antigen receptor-T cell therapy. **: Clinical trials ongoing. *: Preclinical, ↑: activated, ↓: inactivated.
